# The influence of exogenous methyl jasmonate on the structure and physicochemical properties of wheat starch under cadmium stress

**DOI:** 10.3389/fpls.2025.1725845

**Published:** 2025-12-02

**Authors:** Hairong Wang, Dazhong Zhang, Liwen Meng, Fujing Yang, Zhiwei Sun, Yilong Li, Qin Ding, Na Niu, Lingjian Ma

**Affiliations:** 1 College of Agronomy, Northwest A&F University, Yangling, Shaanxi, China; 2 School of Agriculture, Henan Institute of Science and Technology, Xinxiang, Henan, China; 3 College of Horticulture, Northwest A&F University, Yangling, Shaanxi, China

**Keywords:** wheat, grain, cadmium stress, methyl jasmonate, starch

## Abstract

**Introduction:**

MeJA enhances Cd stress resistance and reduces Cd accumulation in wheat (*Triticum aestivum* L.). Our previous study demonstrated that exogenous MeJA regulated wheat responses to Cd stress in a concentration-dependent manner. Building on this, the present study investigated the effects of MeJA spraying on grain weight and starch physicochemical properties in wheat under Cd stress.

**Methods:**

Wheat plants were exposed to different soil Cd concentrations (0, 5, 50 mg/kg) and treated with foliar sprays of MeJA at varying concentrations (0, 1 and 10 µM).

**Results:**

Cd stress significantly reduced grain weight, inhibited starch synthesis, and impaired starch physicochemical properties by decreasing crystallinity, gelatinization enthalpy, and freeze-thaw stability. In contrast, low-concentration MeJA (1 µM) significantly increased thousand-grain weight, total starch content, and B-type starch granule content, while improving starch crystalline structure, thermal stability, and functional properties, thereby alleviating Cd-induced damage. Genotypic variation revealed a MeJA-mediated trade-off between stress defense and developmental metabolism, with the Cd-tolerant cultivar exhibiting a more efficient jasmonate signaling and metabolic compensation mechanism.

**Discussion:**

This study extends previous physiological findings to the starch quality level and provides new mechanistic insight into MeJA-mediated regulation of grain quality and stress adaptation in wheat grown under Cd-contaminated conditions.

## 1 Introduction

Wheat is one of the three major food crops in the world and is recognized for its rich nutritional components. It is commonly processed into foods such as noodles, bread, and biscuits, which are consumed by numerous people worldwide. In China, per capita annual wheat consumption is projected to increase by 13%−18% by 2030 and 28%−50% by 2050 (Mottaleb et al., 2022). Starch is the predominant component of the wheat grains, accounting for approximately 70% of the grain dry weight, and exhibits a positive correlation with grain weight (Yuan et al., 2023). The molecular composition and structural organization of starch strongly affect the quality and functional performance of wheat-based products (Ghodke et al., 2009; Mulla et al., 2010). Specifically, the physicochemical properties of wheat starch, including granule size distribution, relative crystallinity, swelling power, and gelatinization behavior, serve as pivotal determinants of grain quality, flour processing performance, and end-product quality (Jia et al., 2023).

Currently, heavy metal pollution is a major environmental challenge, with cadmium (Cd) being one of the most widespread contaminants. The average Cd concentration in soils is approximately 0.2 mg/kg (Ali et al., 2020), but anthropogenic activities and industrialization have markedly increased soil Cd levels. In China, approximately 33.54% of farmland and 44.65% of urban soils are contaminated by Cd (Yuan et al., 2021). Plants readily absorb Cd from the soil through their roots and accumulate it in various tissues, including leaves and grains (Jarup and Akesson, 2009; Zhou et al., 2018). This accumulation severely disrupts plant growth and development and often leads to potential transmission through the food chain, posing serious threats to human health. With population growth, dietary exposure to Cd-contaminated food is also increasing (Rizwan et al., 2016). Excessive Cd accumulation in the edible plant parts threatens human health upon consumption, potentially leading to renal dysfunction, osteoporosis, and increased cancer risks (Buha et al., 2019). Wheat is highly susceptible and sensitive to Cd accumulation, and soil Cd concentration as low as 5 mg/kg can reduce wheat yield by more than 10% (Zhang et al., 2023). In rice, Cd accumulation has been proven to significantly disrupt nutrient synthesis pathways in the grains, alter starch accumulation and protein composition, and ultimately damage grain quality (Xue et al., 2014). Moreover, bioavailable Cd content in rice has been positively correlated with the amylose content (Yang et al., 2023). In wheat, Cd stress affects the starch granule size and structure (Zhang X. Q. et al., 2024). Collectively, these studies indicate that Cd stress can negatively affect the grain quality of cereal crops. However, current research has mainly focused on reducing Cd accumulation in plants, while relatively little attention has been paid to its impact on grain quality. Therefore, identifying effective strategies to alleviate Cd stress and mitigate its adverse effects on wheat grain quality is of great importance.

Jasmonates, such as jasmonic acid (JA) and methyl jasmonate (MeJA), are plant hormones that regulate growth and development. These plant growth regulators also participate in responses to abiotic and biotic stresses, play important roles in stress signaling, and are closely associated with plant stress resistance (Kaushik et al., 2024; Ueda et al., 2020). In rice, JA treatment reduces Cd accumulation in the seedlings and decreases Cd translocation from roots to grains, thereby lowering the grain Cd concentration (Li et al., 2022). Similarly, exogenous application of MeJA in tomato plants enhances the antioxidant capacity, inhibits Cd transmembrane transport, and enriches less toxic Cd species, alleviating Cd-induced toxicity (Wei et al., 2022). Our previous study demonstrated that an appropriate concentration of MeJA effectively alleviated the inhibitory effects of Cd on wheat growth and photosynthetic performance by enhancing stomatal conductance, intercellular CO_2_ concentration, and transpiration rate (Zhang D. Z. et al., 2024). These findings highlight the role of MeJA in alleviating Cd toxicity in plants. Moreover, our previous work indicated that appropriate concentrations of MeJA can enhance photosynthetic efficiency, promote carbon assimilation, and facilitate assimilate transport to grains, thereby improving overall plant performance under Cd stress. However, how MeJA affects grain quality traits—particularly starch structure and physicochemical properties—under Cd stress has not been systematically investigated. Clarifying these effects is essential for understanding the hormone-mediated coordination between stress defense and carbohydrate metabolism in cereals.

Therefore, to further explore these effects, we investigated two wheat cultivars with contrasting Cd tolerance, *Zhongyu* 10 (tolerant) and *Luomai* 23 (sensitive). Building on our previous findings that exogenous MeJA alleviates Cd-induced inhibition of photosynthesis and growth, we applied a gradient of MeJA concentrations under different Cd stress levels to examine their effects on thousand-grain weight, starch structural characteristics, and physicochemical properties. This study aims to elucidate how MeJA regulates the balance between defense and carbon allocation under Cd stress, providing a theoretical foundation for improving wheat grain quality and yield stability in Cd-contaminated environments.

## 2 Materials and methods

### 2.1 Materials and experimental design

Two wheat cultivars with differing Cd tolerance, *Zhongyu 10* (ZY10, Cd-tolerant) and *Luomai 23* (LM23, Cd-sensitive), were used as experimental materials. A pot experiment was conducted under sunlight greenhouse conditions simulating field environments (**Table 1**). Seeds were sown in October 2021, and four uniform seedlings were retained per pot. Plants were grown throughout the entire growth period and harvested in May 2022. Soil Cd concentrations were adjusted to 0 (control), 5 (low Cd), and 50 mg/kg (high Cd) using CdCl_2_·2.5H_2_O, which correspond to Cd contamination levels commonly observed in agricultural soils (He, 2012; Zhang et al., 2022). Three MeJA spraying concentrations were applied: 0 μM (distilled water), 1μM, and 10 μM. These concentrations were chosen based on previous reports and our prior studies, in which MeJA effectively alleviated Cd-induced toxicity within this range (Wei et al., 2021; Zhang D. Z. et al., 2024). Foliar spraying was performed once at each of four key growth stages—seedling, jointing, flowering, and grain-filling. Spraying was conducted between 9:00 and 11:00 a.m., ensuring that both adaxial and abaxial leaf surfaces were evenly wetted (approximately 8 mL per plant). Plants receiving the same MeJA concentration were arranged in a randomized block design with three biological replicates. To minimize interference caused by MeJA volatilization, a minimum spacing of 1.5 m was maintained between different MeJA treatments.

**Table 1:** Experimental design.

Variety	Treatment	Sign
ZY10	0 Cd + 0 μM MeJA	Z-0-0 (CK)
	0 Cd + 1 μM MeJA	Z-0-1
	0 Cd + 10 μM MeJA	Z-0-10
	5 mg/kg Cd + 0 μM MeJA	Z-5-0
	5 mg/kg Cd + 1 μM MeJA	Z-5-1
	5 mg/kg Cd + 10 μM MeJA	Z-5-10
	50 mg/kg Cd + 0 μM MeJA	Z-50-0
	50 mg/kg Cd + 1 μM MeJA	Z-50-1
	50 mg/kg Cd + 10 μM MeJA	Z-50-10
LM23	0 Cd + 0 μM MeJA	L-0-0 (CK)
	0 Cd + 1 μM MeJA	L-0-1
	0 Cd + 10 μM MeJA	L-0-10
	5 mg/kg Cd + 0 μM MeJA	L-5-0
	5 mg/kg Cd + 1 μM MeJA	L-5-1
	5 mg/kg Cd + 10 μM MeJA	L-5-10
	50 mg/kg Cd + 0 μM MeJA	L-50-0
	50 mg/kg Cd + 1 μM MeJA	L-50-1
	50 mg/kg Cd + 10 μM MeJA	L-50-10

### 2.2 Determination of thousand-grain weight and starch content

One thousand grains were randomly selected to determine the thousand-grain weight, maintaining triplicate samples for each treatment group. The starch and amylose contents were analyzed using the Total Starch Content Assay Kit and the Amylose Content Assay Kit (Solarbio, Shanghai, China), respectively.

### 2.3 Starch isolation and purification

Starch was extracted from the wheat grains using the methods reported by Wen et al (Wen et al., 2024). and Gao et al (Gao et al., 2022), with slight modifications. Briefly, wheat flour was kneaded into dough and washed with distilled water to extract starch. The obtained starch mixture was then soaked in 0.2% NaOH, allowed to rest at room temperature for 4 h, and centrifuged at 4000 × g for 10 min. The upper layer of impurities was removed by scraping. The centrifugation and scraping steps were repeated until no visible impurities remained. The starch was further washed with anhydrous ethanol, centrifuged at 4000 × g for 10 min at room temperature, and dried in an oven at 35 °C for 48 h. The dried starch was finely ground, sieved through a 100-mesh sieve, and stored at 4 °C for subsequent analysis.

### 2.4 Starch morphology and particle size

The morphology of starch granules in the wheat grains was analyzed using a tabletop scanning electron microscope (TM4000 Plus, HITACHI, Japan). The dried starch sample was fixed on the loading platform of the microscope with double-sided tape, sputter-coated with gold, and observed at 15 kV and 1500x magnification. The starch particle size was measured using a laser diffraction particle size analyzer (SYNC, Microtrac Inc., USA) (Gao et al., 2022).

### 2.5 X-ray diffraction

The relative crystallinity of starch in the wheat grains was determined using an X-ray diffractometer (Miniflex 600, Rigaku, Japan) (Guo et al., 2022). Measurement conditions were set as follows: operating voltage 40 kV, current 100 mA, scanning range 5°–45°, step size 0.02°, and scanning speed 5°/min. The XRD patterns were analyzed using Jade 6 software (Materials Data, Inc., Livermore, CA, USA).

### 2.6 Attenuated total reflectance-fourier transform infrared spectroscopy

The starch samples were analyzed using an attenuated total reflectance-Fourier transform infrared (ATR-FTIR) spectrometer (Nicolet iS10, Thermo Fisher Scientific, USA) (Guo et al., 2018). Spectra were collected at a resolution of 4 cm^−1^ with 32 times over the range 400–4000 cm^−1^. The peak intensity ratios of the 1045/1022 cm^−1^ and 1022/995 cm^−1^ bands were obtained via deconvolution to determine the molecular order of starch.

### 2.7 Differential scanning calorimetry

The thermal properties of starch in the wheat grains were determined using a differential scanning calorimeter (DSC, TA Q2000, TA Instruments, USA) (Wang Q. F. et al., 2022). Approximately 3 mg of starch was weighed, placed in an aluminum crucible, and thoroughly mixed with 9 μL of deionized water (sample:water = 1:3). The crucible was further sealed with a lid and stored at 4°C for 12 h before measurement. The DSC thermogram was obtained for the sample under the following conditions: an empty crucible was used as a control, the heating rate was 10°C/min, the temperature range was 30–110°C, and the nitrogen flow rate was 50 mL/min. The characteristic thermal parameters, including onset temperature (To), peak temperature (Tp), conclusion temperature (Tc), and gelatinization enthalpy (ΔH), were calculated from the thermograms using the TA Universal Analysis software.

### 2.8 Solubility and swelling power

The solubility and swelling power of starch were determined according to the method described by Wang et al (Wang et al., 2010). About 0.2 g of starch (m_1_) was mixed with 10 mL of distilled water and heated at 95 °C for 30 min in a water bath with shaking every 5 min. The mixture was cooled to room temperature and centrifuged at 8000 × g for 10 min. The supernatant was transferred into an aluminum container (m_2_), and both the aluminum container and the precipitated paste remaining in the centrifuge tube were dried in an oven at 105 °C. The weight of the aluminum container with the residue was recorded (m_3_), and the weight of the dry matter in the centrifuge tube was determined (m_4_). The solubility and swelling power were calculated using the following formulas:


$$\text{Solubility }\left(\%\right)=\left(m_{3}-m_{2}\right)/m_{1}\times 100\;$$

$$\text{Swelling power }\left(\text{g}/\text{g}\right)=m_{4}/\left[m_{1}\times \left(100-\text{Solubility}\right)\right]$$

### 2.9 Freeze-thaw stability

A 6% starch suspension was prepared by dissolving 0.45 g of starch in 7.5 mL of distilled water. The suspension was heated at 95 °C for 30 min in a water bath with shaking every 10 min. After cooling to room temperature, the gel was weighed (m_1_) and frozen at -20 °C for 24 h. The paste was then thawed at room temperature and centrifuged at 3000 × g for 15 min. The freezing-thawing-centrifuging-weighing steps were repeated three times, and the supernatant obtained at each stage was collected. The total weight of the supernatants was recorded (m_2_) (Marboh and Mahanta, 2021) and used to calculate the water separation rate as follows:


$$\text{ Water separation rate }\left(\%\right)=m_{2}/m_{1}\times 100$$

### 2.10 Light transmittance

A 1% starch paste was prepared by dissolving 0.1 g of starch obtained from the wheat grains in 10 mL of distilled water. The mixture was heated at 95°C for 30 min with shaking every 5 min. After cooling to room temperature, the paste was allowed to stand for 24 h, and the transmittance at 620 nm was measured using a spectrophotometer (Wang et al., 2023).

### 2.11 Data analysis

All data were expressed as the mean ± standard deviation of three independent replicates. Analysis of variance (ANOVA) was conducted using SPSS 26.0 software (SPSS Inc., USA), and the least significant difference (LSD) test was performed to determine the significant differences between the treatment means (*p* < 0.05). Graphs were plotted using Origin 2025 software.

## 3 Results

### 3.1 The thousand-grain weight and grain starch content of wheat under Cd stress

**Figure 1:**
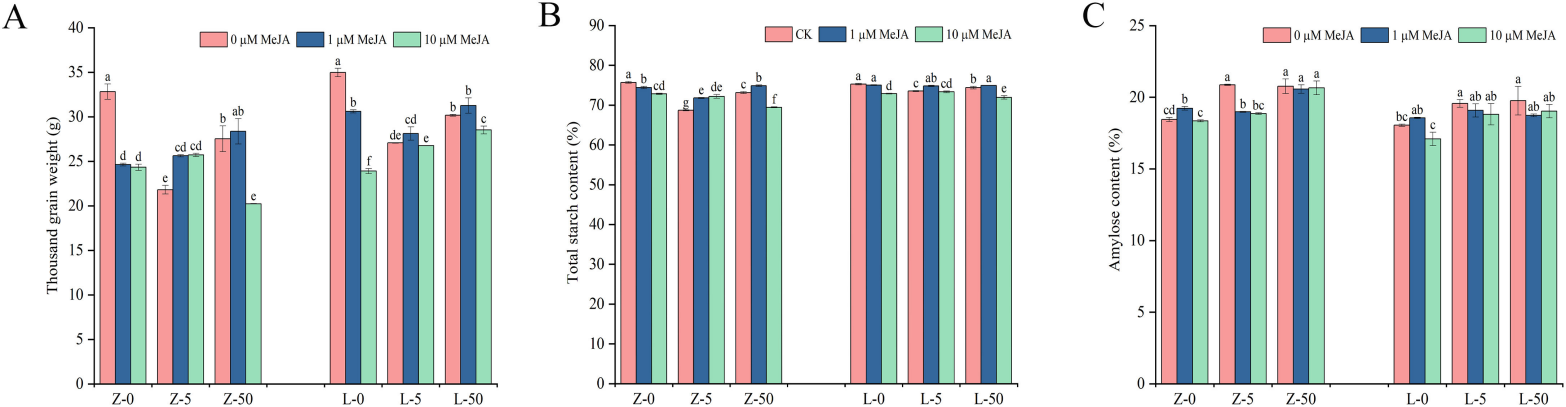
The thousand-grain weight and grain starch content of wheat cultivars under Cd stress and exogenous MeJA treatment. **(A)** thousand-grain weight; **(B)** total starch content; **(C)** amylose content. Different letters above the bars indicate significant differences (LSD; *p* < 0.05) between the treatments of the same cultivar. Z represents ZY10, while L represents LM23. The number following the alphabet Z/L indicates the Cd concentration (mg/kg); “0” denotes 0 mg/kg Cd, “5” denotes 5 mg/kg Cd, and “50” denotes 50 mg/kg Cd.

The thousand-grain weight and grain starch content of ZY10 and LM23 wheat cultivars under different treatments are shown in **Figure 1A**. Compared with the control (CK), both Cd and MeJA treatments significantly affected the thousand-grain weight of the cultivars. Specifically, Cd alone at 5 mg/kg and 50 mg/kg concentrations significantly decreased the thousand-grain weight by 33.55% and 16.15% in ZY10, and by 22.59% and 13.73% in LM23. However, under the same Cd treatments, applying exogenous MeJA at a 1 μM concentration significantly increased the thousand-grain weight by 17.47% and 3.09% in ZY10, and by 3.88% and 3.61% in LM23, relative to the corresponding Cd-treated CK. In contrast, under the 50 mg/kg Cd stress, 10 μM MeJA significantly reduced the thousand-grain weight by 26.49% in ZY10 and 5.47% in LM23 compared with their Cd-treated CK. These results indicated that MeJA modulated wheat grain weight under Cd stress in a concentration-dependent manner. The extent of increase or decrease for each parameter was calculated and is available in the **Supplementary Materials** (**Supplementary Table S1**).

The wheat grains showed significant changes in starch content under Cd and exogenous MeJA treatments (**Figures 1B, C**). Compared with CK, Cd at 5 mg/kg and 50 mg/kg significantly reduced the total starch content in ZY10 by 9.26% and 3.38%, while significantly increasing the amylose content by 13.12% and 12.58%. In LM23, the same Cd treatments significantly reduced the total starch content by 2.32% and 1.16% and significantly increased the amylose content by 8.42% and 9.47%. Notably, applying exogenous MeJA at a 1 μM concentration under Cd stress significantly increased the total starch content in both ZY10 and LM23 grains. Meanwhile, MeJA at both concentrations reduced the amylose content in the grains of both cultivars under 5 mg/kg and 50 mg/kg Cd stress. The extent of increase or decrease for each parameter was calculated and is available in the **Supplementary Materials** (**Supplementary Table S1**).

### 3.2 Starch morphology and particle size

**Figure 2:**
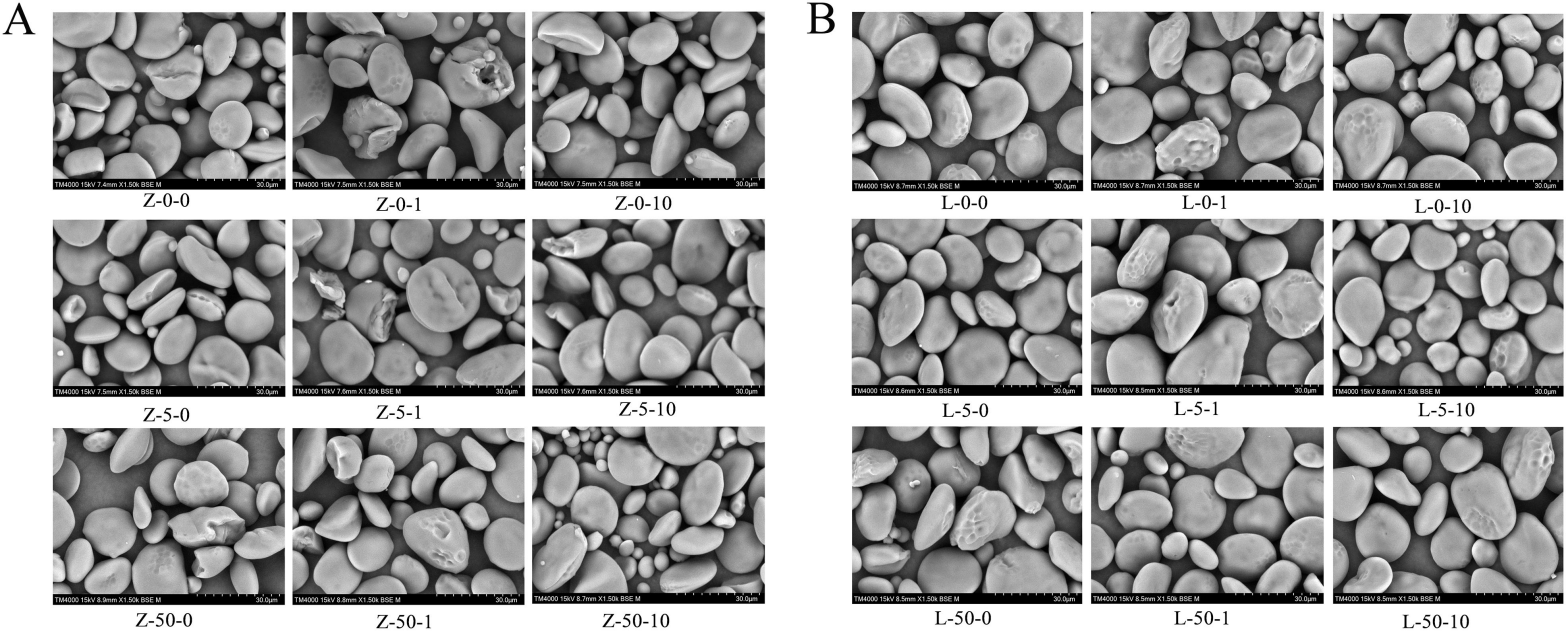
Morphology of the grain starch in wheat cultivars under Cd stress and exogenous MeJA treatment. **(A)** Starch morphology in ZY10; **(B)** Starch morphology in LM23. Z represents ZY10, while L represents LM23. The number following the alphabet L/Z indicates the Cd concentration (mg/kg): “0” denotes 0 mg/kg Cd, “5” denotes 5 mg/kg Cd, and “50” denotes 50 mg/kg Cd.

The morphology of starch in the wheat grains was observed using a scanning electron microscope (**Figure 2**). Compared with CK, treatments with MeJA or Cd increased the starch granule breakage rate and produced more surface craters, indicating that both these chemicals affected the integrity of starch in wheat grains. Under 50 mg/kg Cd stress, spraying 10 μM MeJA significantly increased the proportion of small starch granules in ZY10 (**Figure 2A**). Similarly, under 5 mg/kg Cd stress, spraying 10 μM MeJA significantly increased the proportion of small starch granules in LM23 (**Figure 2B**). Notably, the surfaces of starch granules in the grains of both cultivars appeared smoother after spraying 10 μM MeJA.

Furthermore, the particle size of the starch granules was determined using a laser particle size analyzer (**Table 2**). Compared with CK, treatments with Cd or exogenous MeJA promoted A-type starch in ZY10, resulting in increases in volume mean diameter (D[4,3]), the surface area mean diameter (D[3,2]), and the characteristic particle sizes (D10, D50, D90), while an opposite trend was observed in LM23. Exogenous MeJA at 1 μM concentration significantly increased the proportion of B-type starch granules in both ZY10 and LM23 under Cd stress, leading to smaller starch particles. In addition, exogenous MeJA at 10 μM concentration increased the starch particle size in ZY10 but decreased it in LM23 under 5 mg/kg Cd stress. However, exogenous MeJA at 10 μM concentration decreased the starch particle size in ZY10 and increased it in LM23 under 50 mg/kg Cd stress.

**Table 2:** Particle size of starch granules in the grains of wheat cultivars under Cd stress and exogenous MeJA treatment.

Varieties	Treatments	D[4,3]	D[3,2]	D10	D50	D90	A≥10 μm	B<10 μm
ZY10	Z-0-0	19.01 ± 0.09bc	15.94 ± 0.22d	11.47 ± 0.14d	17.90 ± 0.11bc	27.75 ± 0.12ab	24.56 ± 0.28d	75.44 ± 0.28a
	Z-0-1	19.59 ± 0.31abc	17.08 ± 0.39abcd	12.31 ± 0.30abcd	18.39 ± 0.29abc	28.14 ± 0.36ab	46.94 ± 0.81c	53.07 ± 0.81b
	Z-0-10	19.76 ± 0.63ab	17.41 ± 0.84abcd	12.53 ± 0.63abcd	18.55 ± 0.58ab	28.28 ± 0.71ab	57.60 ± 1.00c	47.41 ± 1.00c
	Z-5-0	19.49 ± 0.09abc	17.67 ± 0.08abc	12.85 ± 0.06abc	18.24 ± 0.08abc	27.31 ± 0.15bc	84.91 ± 0.20a	15.10 ± 0.20e
	Z-5-1	18.96 ± 0.75bc	16.68 ± 0.97bcd	11.98 ± 0.76bcd	17.75 ± 0.69bc	27.14 ± 0.81bc	48.69 ± 0.54c	51.31 ± 0.54b
	Z-5-10	20.44 ± 0.23a	18.51 ± 0.24a	13.41 ± 0.20a	19.14 ± 0.23a	28.80 ± 0.30a	86.23 ± 0.94a	13.78 ± 0.94e
	Z-50-0	20.02 ± 0.23ab	18.06 ± 0.22ab	13.02 ± 0.19ab	18.71 ± 0.23ab	28.32 ± 0.30ab	84.43 ± 0.86a	15.58 ± 0.86e
	Z-50-1	18.91 ± 0.37bc	16.74 ± 0.50bcd	11.91 ± 0.39bcd	17.68 ± 0.35bc	27.11 ± 0.38bc	66.27 ± 0.97b	33.74 ± 0.97d
	Z-50-10	18.47 ± 0.41c	16.18 ± 0.65cd	11.70 ± 0.45cd	17.33 ± 0.36c	26.35 ± 0.42c	50.34 ± 0.80c	49.66 ± 0.80b
LM23	L-0-0	22.95 ± 0.36a	20.82 ± 0.36a	15.13 ± 0.31a	21.46 ± 0.35a	32.20 ± 0.42a	93.20 ± 0.44b	6.80 ± 0.44c
	L-0-1	21.31 ± 0.32cd	18.88 ± 0.44bcd	13.64 ± 0.28cd	19.93 ± 0.28cd	30.26 ± 0.39bcd	78.66 ± 0.35d	21.35 ± 0.35a
	L-0-10	20.91 ± 0.63cde	18.82 ± 0.68bcd	13.65 ± 0.50cd	19.60 ± 0.52cde	29.48 ± 0.77cd	86.45 ± 0.55c	13.55 ± 0.55b
	L-5-0	21.75 ± 0.32bc	19.79 ± 0.31ab	14.44 ± 0.26b	20.34 ± 0.30bc	30.39 ± 0.41bc	93.23 ± 0.20b	6.78 ± 0.20c
	L-5-1	21.46 ± 0.15cd	19.35 ± 0.14bc	14.01 ± 0.11bc	20.02 ± 0.14cd	30.19 ± 0.23bcd	86.89 ± 0.20c	13.11 ± 0.20b
	L-5-10	20.63 ± 0.71de	18.60 ± 0.70cd	13.45 ± 0.55cd	19.30 ± 0.63de	29.11 ± 0.90de	86.94 ± 0.46c	13.07 ± 0.46b
	L-50-0	21.70 ± 0.23bc	19.79 ± 0.31ab	14.47 ± 0.29b	20.33 ± 0.31bc	30.28 ± 0.25bcd	93.09 ± 0.87b	6.91 ± 0.87c
	L-50-1	20.99 ± 0.15cd	19.02 ± 0.14bcd	13.81 ± 0.11c	19.7 ± 0.14cd	29.52 ± 0.19cd	86.74 ± 0.31c	13.26 ± 0.31b
	L-50-10	22.57 ± 0.28ab	20.71 ± 0.27a	15.32 ± 0.24a	21.11 ± 0.27ab	31.16 ± 0.35ab	98.54 ± 0.17a	1.47 ± 0.17d

D[4,3]: Volume-weighted mean diameter of starch granules, representing the mean particle size based on volume distribution. D[3,2]: Surface-weighted mean diameter, calculated from the surface area distribution of particles. D10, D50, and D90: Characteristic percentile diameters at which 10%, 50%, and 90% of the total starch granule population (by volume) fall below these size values, respectively. A, A-type starch granules (≥10 μm). B, B-type starch granules (<10 μm). Different letters indicate significant differences (LSD; *p* < 0.05) among the different treatments of the same cultivar.

### 3.3 X-ray diffraction

**Figure 3:**
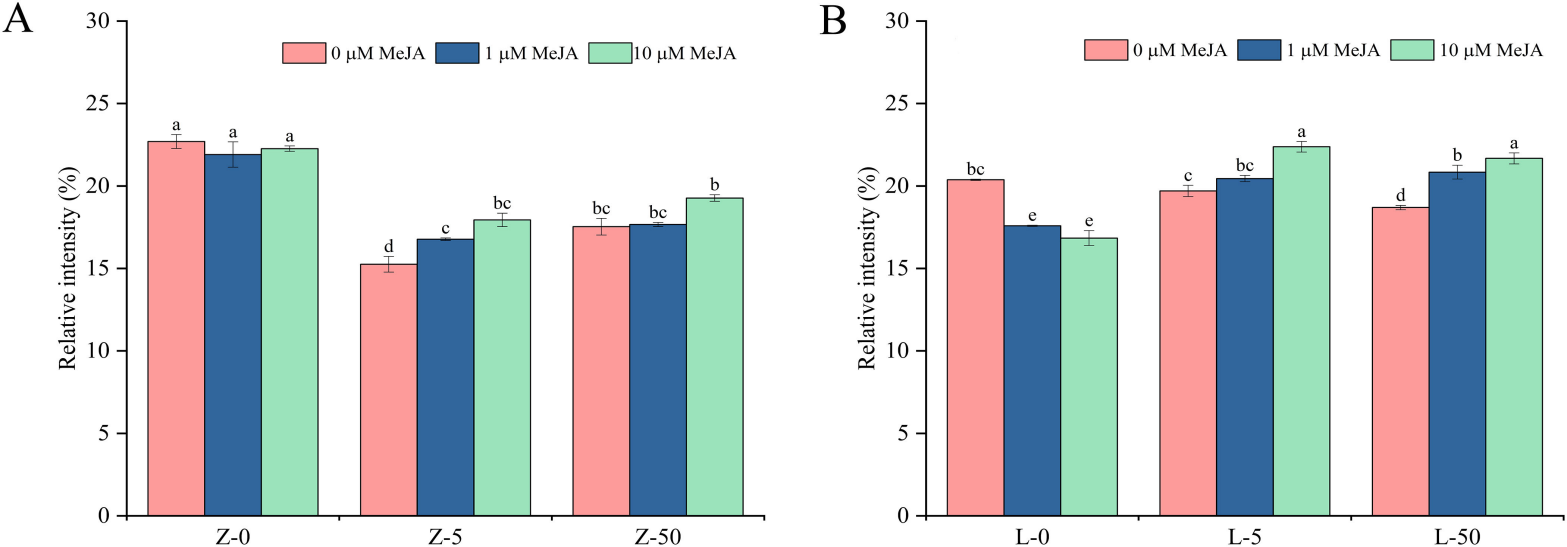
Relative crystallinity of starch in the grains of wheat cultivars under Cd stress and exogenous MeJA treatment. **(A, B)** show the relative crystallinity of ZY10 and LM23 starch, respectively. Different lowercase letters above the bars indicate significant differences between treatments of the same cultivar (LSD; *p* < 0.05). Z represents ZY10, while L represents LM23. The number following the alphabet L/Z indicates the Cd concentration (mg/kg): “0” denotes 0 mg/kg Cd, “5” denotes 5 mg/kg Cd, and “50” denotes 50 mg/kg Cd.

**Supplementary Figure S1** shows the X-ray diffractograms of starch in the wheat grains. All starch samples exhibited distinct diffraction peaks at 15°, 17°, 18°, and 23°, characteristic of the typical A-type crystal structure. However, the treatments affected the starch crystallinity in the wheat grains (**Figures 3A, B**). Compared with CK, Cd treatment alone significantly reduced the starch crystallinity in both cultivars, with a greater reduction observed in ZY10. Similarly, treatment with exogenous MeJA alone reduced starch crystallinity, particularly in LM23. These results proved that Cd and MeJA reduced the crystallinity of starch. However, exogenous MeJA tended to increase the starch crystallinity of ZY10 and LM23 under Cd stress, showing an overall rising trend with increasing MeJA concentration, although some changes were not statistically significant. Under 5 mg/kg and 50 mg/kg Cd stress, spraying 10 μM MeJA significantly increased the crystallinity by 17.63% and 9.86% in ZY10, and by 13.60% and 15.94% in LM23, respectively. The extent of increase or decrease for each parameter was calculated and is available in the **Supplementary Materials** (**Supplementary Table S2**).

### 3.4 ATR-FTIR spectrum

**Figure 4:**
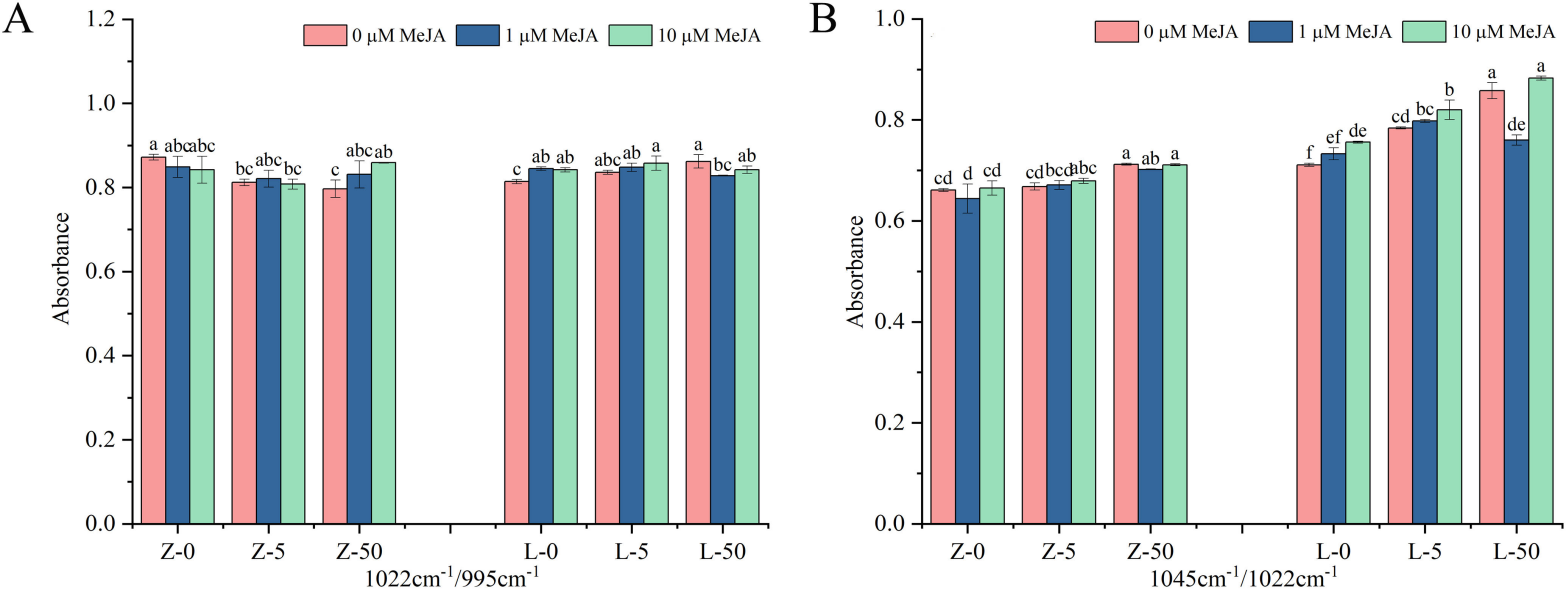
Effects of Cd stress and exogenous MeJA treatment on the FTIR of wheat grain starch. **(A, B)** show the infrared ratios of starch. Z represents ZY10, while L represents LM23. The number following the alphabet L/Z indicates the Cd concentration (mg/kg): “0” denotes 0 mg/kg Cd, “5” denotes 5 mg/kg Cd, and “50” denotes 50 mg/kg Cd.

To investigate the effects of different treatments on the short-range molecular order of wheat starch, the 800−1200 cm^−1^ spectral region was analyzed. The spectral deconvolution of this region is shown in **Supplementary Figure S2** The peak intensities of each starch sample under different treatments were similar, indicating that Cd and exogenous MeJA did not noticeably affect the positions of the peaks. Cd stress and exogenous MeJA treatment both decreased the 1022 cm^−1^/995 cm^−1^ ratio in ZY10 but increased it in LM23 (**Figure 4A**). Under 50 mg/kg Cd stress, the 1045 cm^−1^/1022 cm^−1^ ratio increased significantly by 7.72% in ZY10 and 20.68% in LM23 compared with CK, indicating a substantial impact of Cd on starch molecular order (**Supplementary Table S2**). Notably, applying exogenous MeJA under 5 mg/kg Cd stress generally showed a trend of increasing the 1045 cm^−1^/1022 cm^−1^ ratio, with the ratio in LM23 significantly increasing with higher MeJA concentrations. However, under 50 mg/kg Cd stress, 1 μM MeJA tended to decrease this ratio in both cultivars, reaching significance in LM23; while 10 μM MeJA increased it (**Figure 4B**).

### 3.5 Thermal properties of starch

DSC was used to evaluate the thermal properties of grain starch (**Supplementary Figure S3**), and the thermodynamic parameters are presented in **Table 3**. Significant differences in gelatinization temperatures were observed between cultivars. The onset temperatures (To) ranged from 55.12°C to 57.59°C for ZY10 and from 52.56°C to 55.41°C for LM23; the peak temperatures (Tp) ranged from 61.21°C to 62.42°C for ZY10 and from 58.85°C to 60.73°C for LM23; the end temperatures (Tc) ranged from 73.94°C to 76.25°C for ZY10 and from 69.04°C to 72.00°C for LM23. Compared with CK, Cd, and exogenous MeJA treatments alone significantly decreased the enthalpy (ΔH) of ZY10 and LM23 (**Table 3**). In contrast, applying exogenous MeJA under Cd stress significantly increased the ΔH of both cultivars, with higher MeJA concentrations producing greater increases. These ΔH trends were consistent with the observed changes in starch crystallinity (**Figures 3C, D**). Under 5 mg/kg Cd stress, ΔH in both ZY10 and LM23 showed a significant upward trend with increasing MeJA concentration. Under 50 mg/kg Cd stress, 1 μM MeJA significantly increased ΔH in LM23 but not in ZY10, while 10 μM MeJA significantly increased ΔH in ZY10 but not in LM23.

**Table 3:** DSC parameters of starch in the grains of wheat under Cd stress and exogenous MeJA treatment.

Varieties	Treatments	To	Tp	Tc	ΔH
ZY10	Z-0-0	57.59 ± 0.01a	62.42 ± 0.01a	74.98 ± 0.14b	10.42 ± 0.10a
	Z-0-1	56.81 ± 0.09b	62.2 ± 0.10ab	73.94 ± 0.69c	9.58 ± 0.01d
	Z-0-10	56.07 ± 0.12c	62.29 ± 0.16a	75.93 ± 0.42a	10.15 ± 0.09b
	Z-5-0	56.94 ± 0.06b	62.01 ± 0.05bc	76.25 ± 0.14a	9.64 ± 0.19d
	Z-5-1	56.73 ± 0.08b	61.51 ± 0.01d	75.06 ± 0.12b	9.95 ± 0.03bc
	Z-5-10	56.80 ± 0.33b	62.43 ± 0.18a	75.87 ± 0.36a	10.07 ± 0.07b
	Z-50-0	56.32 ± 0.20c	61.82 ± 0.25c	74.03 ± 0.18c	9.65 ± 0.22d
	Z-50-1	56.08 ± 0.06c	61.55 ± 0.16d	74.79 ± 0.39b	9.76 ± 0.13cd
	Z-50-10	55.12 ± 0.11d	61.21 ± 0.04e	74.97 ± 0.03b	9.94 ± 0.28bc
LM23	L-0-0	54.19 ± 0.01bc	59.73 ± 0.07d	70.42 ± 0.48b	10.48 ± 0.24b
	L-0-1	54.47 ± 0.06bc	60.49 ± 0.01ab	70.81 ± 0.32b	9.38 ± 0.40e
	L-0-10	52.56 ± 1.26d	59.72 ± 0.36d	70.87 ± 0.17b	10.18 ± 0.12bc
	L-5-0	54.48 ± 0.13bc	60.17 ± 0.03bc	70.65 ± 0.73b	9.92 ± 0.27cd
	L-5-1	54.71 ± 0.07ab	60.53 ± 0.24a	71.17 ± 0.67ab	10.54 ± 0.01b
	L-5-10	55.41 ± 0.05a	60.73 ± 0.06a	72.00 ± 0.91a	11.09 ± 0.11a
	L-50-0	54.09 ± 0.04bc	59.71 ± 0.38d	69.22 ± 0.75c	9.68 ± 0.13de
	L-50-1	53.81 ± 0.24c	59.95 ± 0.08cd	71.41 ± 0.07ab	11.17 ± 0.26a
	L-50-10	52.78 ± 0.04d	58.85 ± 0.01e	69.04 ± 0.41c	10.00 ± 0.01cd

Different lowercase letters indicate significant differences between treatments of the same cultivar (LSD; *p* < 0.05).

### 3.6 Solubility and swelling power

**Figure 5:**
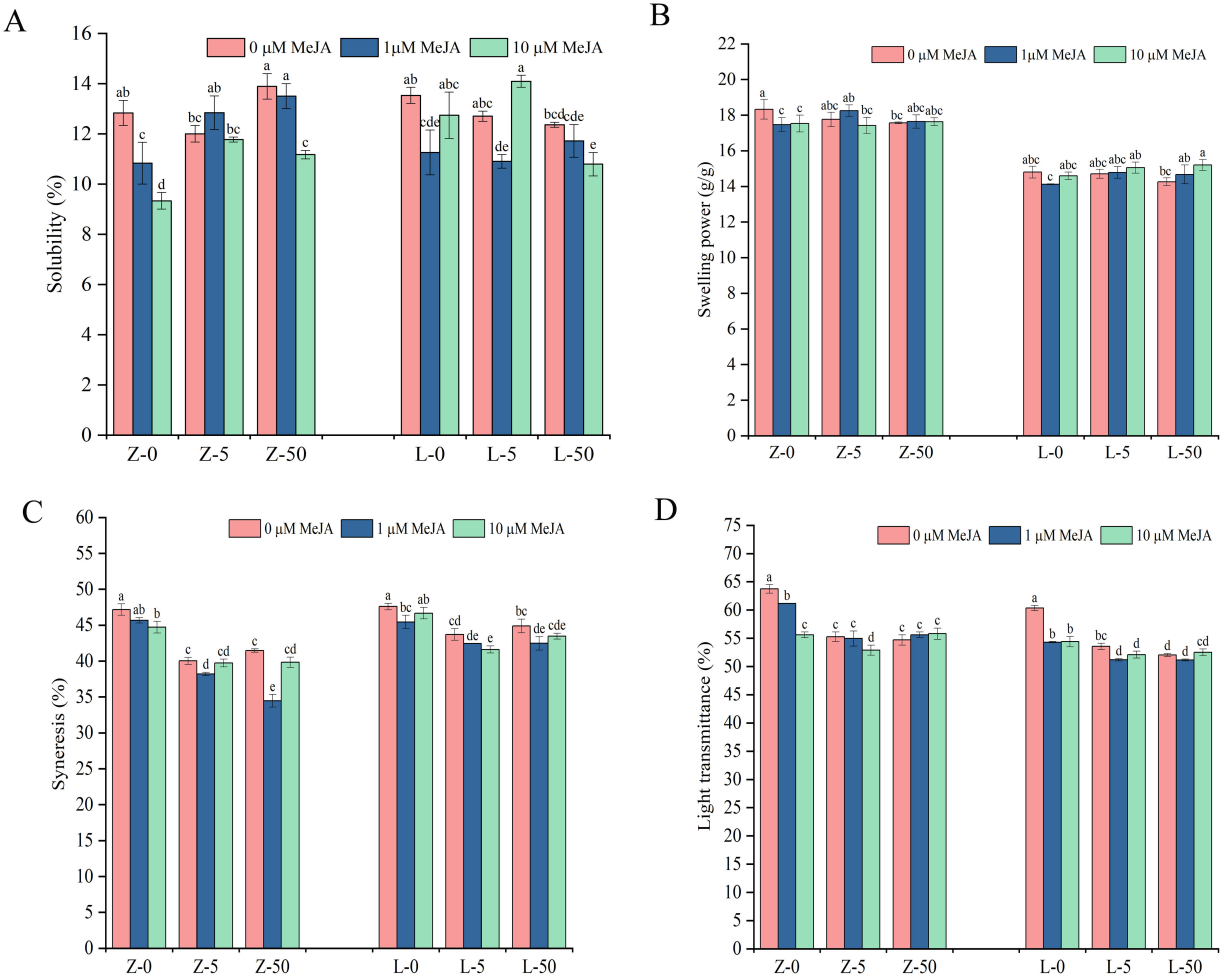
The effect of Cd stress and exogenous MeJA on wheat starch solubility, swelling power, syneresis, and light transmittance. **(A)** Starch solubility; **(B)** Starch swelling power; **(C)** Starch syneresis; **(D)** Light transmittance. Different lowercase letters above the bars indicate significant differences between treatments of the same cultivar (LSD; *p* < 0.05). Z represents ZY10, while L represents LM23. The number following the alphabet L/Z indicates the Cd concentration (mg/kg): “0” denotes 0 mg/kg Cd, “5” denotes 5 mg/kg Cd, and “50” denotes 50 mg/kg Cd.

As shown in **Figures 5A, B**, the swelling power of starch after heating at 90°C ranged from 17.47 to 18.33 g/g in ZY10 and from 14.12 to 15.20 g/g in LM23 (**Supplementary Table S3**). This observation indicated a significant difference in swelling power between the two cultivars. Compared with CK, MeJA and Cd treatments alone tended to reduce the swelling power and solubility in ZY10 and LM23 grains. Notably, applying exogenous MeJA under Cd stress increased the swelling power of ZY10 and LM23 starch. Under 50 mg/kg Cd stress, increasing MeJA concentration led to a gradual decrease in starch solubility and an increase in swelling power in both cultivars. In contrast, under 5 mg/kg Cd stress, exogenous MeJA exhibited inconsistent trends in the two cultivars.

### 3.7 Freeze-thaw stability of starch

The freeze-thaw stability of starch in the wheat grains typically reflects its water-holding capacity, with higher syneresis indicating poorer stability. In this study, compared with CK, Cd and MeJA treatments both significantly reduced the syneresis of starch in ZY10 and LM23, thereby improving freeze-thaw stability (**Figure 5C**). Applying exogenous MeJA under Cd stress further enhanced this effect, with 1 μM MeJA showing a more significant improvement. Specifically, spraying 1 μM MeJA under 5 mg/kg Cd stress reduced the syneresis of starch by 4.57% (significant) in ZY10 and by 2.88% (non-significant) in LM23; under 50 mg/kg Cd stress, the reductions were 16.84% and 5.39%, respectively, both significant (**Supplementary Table S3**).

### 3.8 Light transmittance

The light transmittance of starch under different treatments is shown in **Figure 5D**. Compared with CK, Cd stress and exogenous MeJA treatment both significantly reduced the light transmittance. Notably, applying exogenous MeJA under 5 mg/kg Cd stress further decreased the light transmittance, The application of 10 μM MeJA resulted in significant reductions of 4.25% in ZY10 and 2.71% in LM23 (**Supplementary Table S3**).

## 4 Discussion

The present study systematically investigated the effects of Cd stress (0, 5, and 50 mg/kg) and MeJA application (0, 1, and 10 μM) on grain weight, starch structure, and physicochemical properties of wheat. The results demonstrated that Cd stress significantly inhibited material accumulation, as reflected by reduced grain weight and total starch content, and adversely affected starch physicochemical properties. Nevertheless, exogenous MeJA treatment partially mitigated these Cd-induced detrimental effects.

### 4.1 Effects of exogenous MeJA on grain weight and starch content in wheat under Cd stress

Our previous study demonstrated that the effects of exogenous MeJA on wheat under Cd stress were strongly concentration-dependent. At a low concentration (1 μM), MeJA significantly improved stomatal conductance, intercellular CO_2_ concentration, and transpiration rate, thereby enhancing the formation of photosynthetic assimilates and their translocation to grains. In contrast, a high concentration (10 μM) showed weaker alleviating effects on photosynthesis and markedly reduced the transpiration rate, thereby restricting the transport of water and assimilates to grains (Zhang D. Z. et al., 2024). In the present study, MeJA exhibited distinct effects on grain weight under Cd stress: low-concentration MeJA (1 μM) increased grain weight and total starch content under Cd stress, whereas high-concentration MeJA (10 μM) decreased both parameters (**Table 2**). The parallel changes in starch and grain weight indicate that MeJA regulates carbon partitioning between defense and growth in a concentration-dependent manner. Previous reports have shown that excessive MeJA can inhibit spike development and grain filling (Kim et al., 2009), may be explained by the fact that high MeJA concentrations decrease stomatal conductance and transpiration rate and accelerate leaf senescence (Chen et al., 2013; Yan et al., 2013). Conversely, low MeJA concentrations have been shown to enhance photosynthetic efficiency under heavy metal stress (Per et al., 2016). Consistent with this, in the present study, 10 μM MeJA appeared to suppress photosynthesis, thereby limiting starch biosynthesis under Cd stress and negatively affecting yield formation. In contrast, 1 μM MeJA effectively balanced defense activation with assimilate allocation, maintaining starch biosynthetic activity and improving grain filling efficiency.

Overall, these results suggested that low-concentration MeJA helps maintain a “defense–development” equilibrium under Cd stress, optimizing resource distribution and improving both yield and starch quality. This concentration-dependent response represents a potential strategy for enhancing wheat adaptation to heavy metal stress through fine hormonal regulation.

### 4.2 Effects of exogenous MeJA on starch morphology and granule size of wheat under Cd stress

Starch in the grains is typically characterized by two categories of granules: large A-type starch granules (disc-shaped, ≥10 μm) and small B-type starch granules (spherical,<10 μm) (Yan and Lu, 2020). In both ZY10 and LM23 cultivars, we observed the characteristic A-type and B-type starch granules under control and treated conditions, consistent with previous reports. These findings suggest that neither Cd treatment nor MeJA application altered the fundamental morphology of starch granules. In wheat, the A-type starch begins to form approximately 4 days after anthesis, whereas B-type starch forms later, generally around 12 days after anthesis (Wei et al., 2010). Notably, under Cd stress, exogenous 1 μM MeJA consistently and significantly increased the proportion of B-type starch granules in both cultivars. This suggests that low-concentration MeJA may interfere with the A-type starch formation, thereby promoting the B-type starch granule production and reducing overall starch granule size. Since B-type starch granules differ from A-type granules in gelatinization temperature, digestibility, and dough-processing performance, this shift may have substantial effects on grain physicochemical properties and food processing quality (Guo et al., 2022). This shift may represent an adaptive mechanism to maintain starch synthesis efficiency under stress. Overall, these findings highlight a potential regulatory role of low MeJA concentration in starch granule formation under Cd stress, linking hormonal signaling to starch structural adaptation.

### 4.3 Effects of exogenous MeJA on starch structure of wheat under Cd stress

X-ray diffraction (XRD) is commonly used to detect the crystalline configuration and crystallinity of starch (Zhang Y. T. et al., 2024). In this study, XRD analysis showed that neither Cd stress nor exogenous MeJA altered the A-type crystalline structure of starch in the wheat grains. This observation is consistent with previous studies, indicating that exogenous hormones do not influence the crystalline structure of starch (Ran et al., 2020; Wen et al., 2024). However, MeJA application under Cd stress significantly increased starch crystallinity, particularly at 10 μM, indicating a partial recovery of the crystalline order disrupted by Cd toxicity. In both wheat varieties, starch crystallinity exhibited an opposite trend to amylose content (**Figure 1**). Although the decrease in amylose content was relatively minor, such subtle changes may still reflect delicate alterations in starch composition—specifically, variations in the content and structure of amylopectin. Previous studies have shown that as the major crystalline component of starch, amylopectin contributes to higher crystallinity when its long-chain proportion increases, whereas amylose tends to reduce crystallinity (Tao et al., 2016). We speculate that low concentrations of MeJA may promote the elongation or proper branching of amylopectin chains, thereby restoring crystalline organization and enhancing starch structural integrity under Cd stress. Future studies should further investigate the fine structural changes in amylopectin.

FTIR further provides insight into starch short-range molecular order, particularly in the 800–1200 cm^−1^ spectral region (Sevenou et al., 2002). The absorption peak at 1022 cm^−1^ represents the amorphous region of starch, while the 1045 cm^−1^ peak reflects ordered structures (Guo et al., 2018). In this study, under different concentrations of Cd, the 1045 cm^−1^/1022 cm^−1^ ratio in starch granules increased under the high-concentration (10 μM) MeJA compared with the low-concentration (1 μM) MeJA group; this observation was consistent with the changes in starch crystallinity. (**Figures 3C, D**). These findings indicate that high concentrations of MeJA promote the rearrangement of starch molecular chains and the formation of ordered structures under Cd stress, thereby enhancing starch crystallinity. Because starch crystallinity strongly influences gelatinization, digestibility, and final food processing quality (Jia et al., 2023), this result suggests that MeJA not only alleviates the adverse effects of Cd stress but may also improve grain quality by modulating starch molecular structure.

### 4.4 Impact of exogenous MeJA on functional properties of starch in wheat under Cd stress

The thermal parameters To, Tp, and Tc reflect the quality of starch crystals in the grain, while the ΔH represents the energy required to disrupt the double-helical structure of starch (Shevkani et al., 2016). However, spraying MeJA under Cd stress significantly increased the ΔH in both cultivars, likely due to enhanced starch crystallinity (**Figure 3**). A higher ΔH indicates that more energy is needed for starch pasting, implying a more compact and stable starch crystalline structure, as greater energy input is necessary to disrupt the molecular order and break the hydrogen bonds within the crystalline regions (Zou et al., 2020). This indicates that MeJA partially restored the crystalline arrangement disrupted by Cd toxicity, leading to enhanced starch stability.

Starch swelling power and solubility are temperature-dependent processes affected by granule structure and amylose/amylopectin ratio (Gong et al., 2017). The present study indicates significant genotypic differences in starch solubility and swelling capacity between ZY10 and LM23 (**Figures 5A, B**). These variations probably originated from the inherent differences in starch granule crystalline structure or amylose/amylopectin ratio between the two cultivars (Ran et al., 2020). Under 50 mg/kg Cd stress, exogenous MeJA exhibited concentration-dependent regulatory effects on both cultivars. The reduced solubility may be attributed to MeJA-induced increases in starch molecular cross-linking, whereas the enhanced swelling capacity suggests that MeJA helps preserve water-holding capacity by mitigating Cd-induced damage to starch granule structure. The study also found differences in the response to MeJA under low Cd (5 mg/kg) stress between the two cultivars, which may be associated with starch molecular weight, amylose/amylopectin ratio, and starch granule size (Li et al., 2013; Xu et al., 2018). Overall, the contrasting patterns of solubility and swelling under different treatments further highlight the role of multiple factors in regulating the physicochemical properties of starch.

Similarly, freeze-thaw stability, an important property of starch, reflects its water-holding capacity and affects its edible texture. The higher the syneresis, the poorer the freeze-thaw stability of starch (Ye et al., 2016). Under Cd stress, spraying 1 μM MeJA significantly improved the freeze–thaw stability of both cultivars, enhancing the water-holding capacity of starch paste. The trend of syneresis under Cd stress was consistent with amylose content in wheat (**Table 2**), suggesting that MeJA may indirectly affect starch retrogradation by regulating molecular rearrangement (Marboh and Mahanta, 2021).

Meanwhile, the light transmittance of starch after heating and gelatinization reflects transparency, which affects appearance, processing, and utilization (Li et al., 2016; Wang J. L. et al., 2022). In this study, Cd stress significantly reduced starch light transmittance in both cultivars, indicating impaired starch gelatinization and altered physicochemical properties. Exogenous MeJA treatments further modulated this response in a concentration-dependent manner. Under low Cd stress (5 mg/kg), MeJA (1 μM and 10 μM) slightly decreased starch light transmittance, whereas under high Cd stress (50 mg/kg), 10 μM MeJA partially improved transmittance. This property is typically correlated with starch characteristics such as granule size, amylose content, and swelling degree (Gao et al., 2022; Tao et al., 2021), further supporting the fact that multiple factors influence starch light transmittance. Overall, while Cd stress strongly reduces starch transparency, low-concentration MeJA exhibits a modest ability to modulate this property, highlighting its potential in alleviating heavy metal-induced alterations in starch quality.

### 4.5 Genotype-specific effects of MeJA on starch structure and quality under Cd stress

Previous results showed that exogenous MeJA reduced the root-to-grain Cd translocation coefficient in both ZY10 and LM23, with a stronger inhibitory effect in ZY10. Under 5 mg/kg Cd stress, 10 μM MeJA significantly lowered grain Cd content by 36.1% in LM23 and 39.9% in ZY10 (Zhang D. Z. et al., 2024), indicating that the tolerant cultivar more effectively limits Cd accumulation, thereby protecting starch biosynthesis and grain filling.

Consistent with these findings, the present study revealed that ZY10 exhibited a greater reduction in grain weight and more severe starch structural disruption under Cd stress, suggesting a trade-off strategy that prioritizes antioxidant defense and detoxification at the expense of grain filling. Exogenous MeJA alleviated these effects and enhanced grain weight and starch accumulation in both cultivars, consistent with the role of MeJA in regulating starch biosynthesis and stress adaptation in cereals (Meng et al., 2023). However, ZY10 responded more strongly to MeJA treatment and showed greater sensitivity to concentration changes, implying a more efficient jasmonate signaling and metabolic compensation mechanism.

Notably, MeJA exhibited a concentration-dependent effect. While high MeJA concentration (10 μM) effectively reduced grain Cd content, it also compromised grain quality by decreasing grain weight and starch content—likely due to reduced photosynthetic efficiency and inhibition of key starch-synthesizing enzymes during grain filling (Huang et al., 2021; Kang et al., 2013). We propose that under “Cd + low-concentration MeJA” conditions, ZY10 can coordinate Cd detoxification and starch biosynthesis more effectively through its robust jasmonate signaling network, whereas LM23 shows a weaker response. Future work should focus on the activities and expression patterns of key starch biosynthetic enzymes (AGPase, SSS, GBSS, SBE) to elucidate the genotype-specific regulation under Cd and MeJA treatments.

## 5 Conclusions

The present study reveals a core mechanism governing wheat grain quality under Cd stress—a MeJA-mediated trade-off between defense and development. Low-concentration MeJA (1 μM) optimizes resource allocation, mitigating Cd-induced inhibition of grain filling while enhancing starch structure and functional properties. In contrast, high-concentration MeJA (10 μM) disrupts this balance and thus exhibits a threshold-dependent response. Moreover, the Cd-tolerant cultivar (ZY10) showed a more efficient coordination between Cd detoxification and starch biosynthesis than the Cd-sensitive cultivar (LM23), suggesting genotype-specific differences in jasmonate signaling and metabolic compensation. These findings highlight MeJA as an endogenous regulator that fine-tunes assimilate partitioning and starch metabolism to support wheat adaptation to Cd stress. This work provides a theoretical basis for applying phytohormones to optimize the defense–development balance in wheat under heavy metal stress. Future studies should integrate transcriptomic, metabolomic, and enzyme activity analyses to elucidate the underlying genotype-dependent regulatory network.

## Data Availability

The original contributions presented in the study are included in the article/**Supplementary Material**. Further inquiries can be directed to the corresponding authors.
