# Supplementary material for: The influence of exogenous methyl jasmonate on the structure and physicochemical properties of wheat starch under cadmium stress
Source: Front Plant Sci. 2025 Dec 2;16:1725845. doi: 10.3389/fpls.2025.1725845 (PMC12750620; doi:10.3389/fpls.2025.1725845)
Supplement: Supplementary file 1 [file Supplementaryfile1.docx]

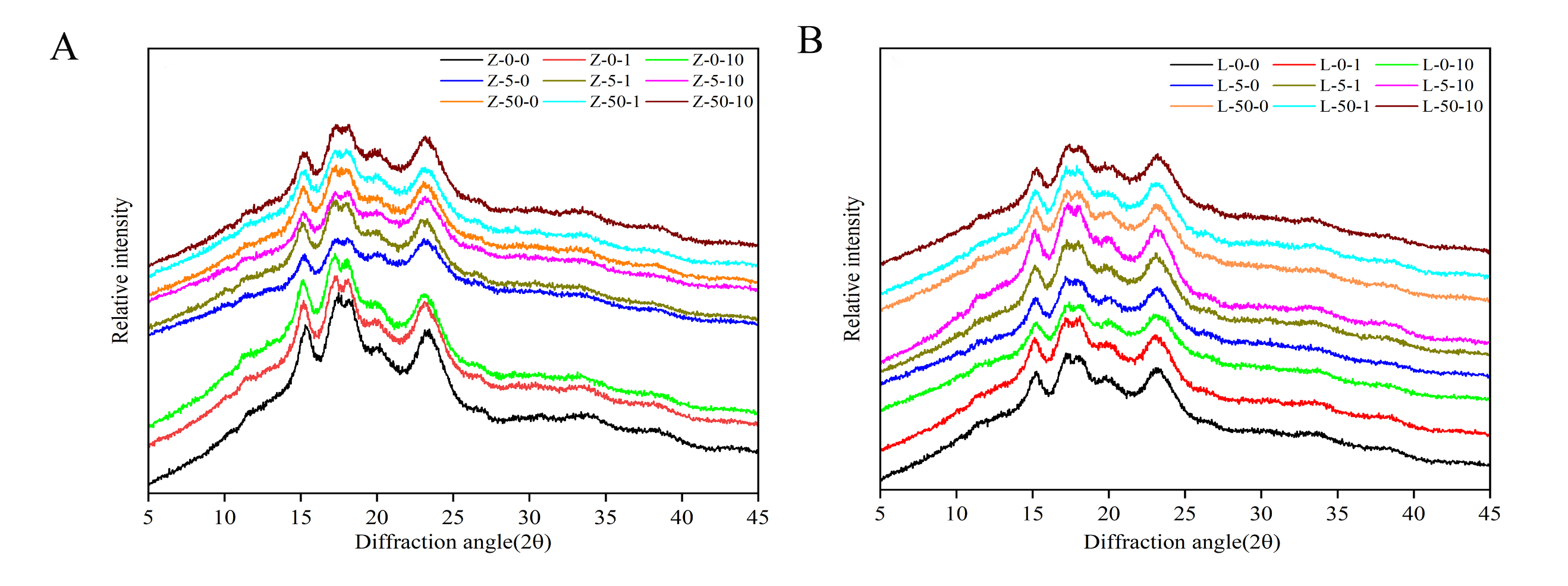


Supplementary Figure 1 X-ray diffractograms of starch in the grains of wheat cultivars under Cd stress and exogenous MeJA treatment. A and B show the XRD diffraction patterns of ZY10 and LM23 starch, respectively.


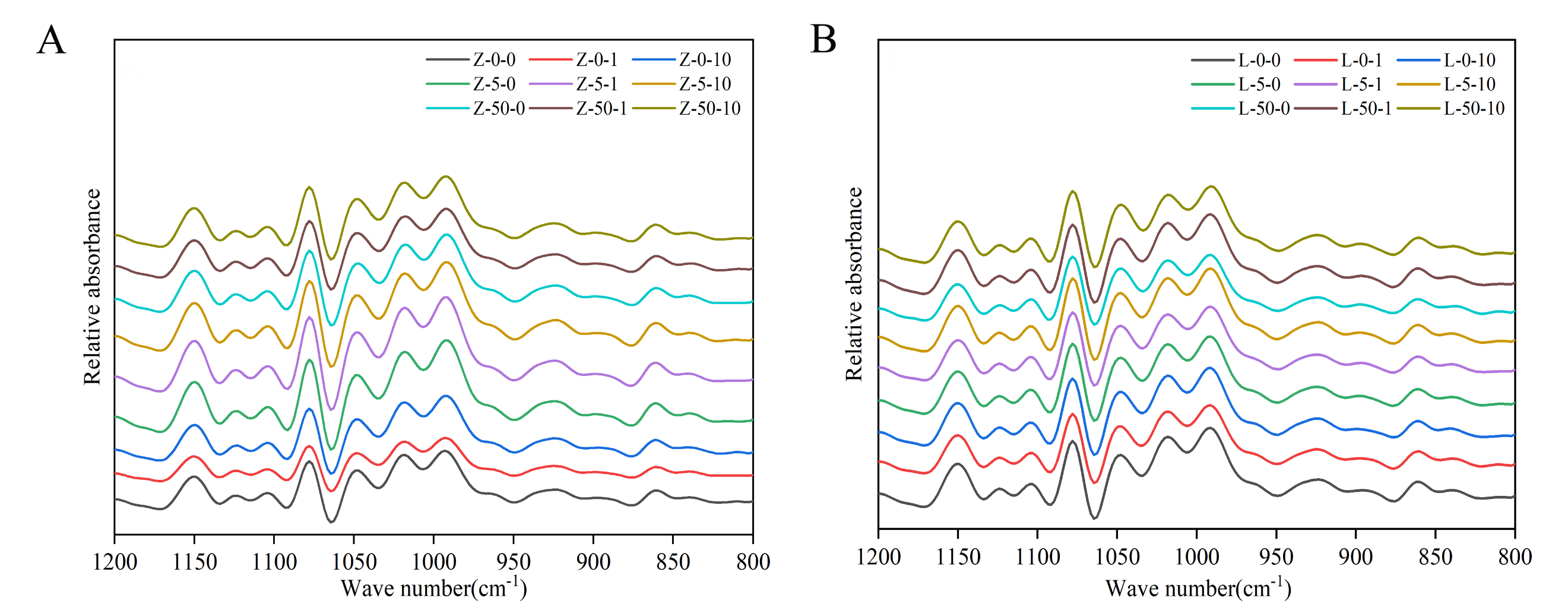


Supplementary Figure 2 Effects of Cd stress and exogenous MeJA treatment on the FTIR of wheat grain starch. A and B show the FTIR spectra of ZY and LM23, respectively.


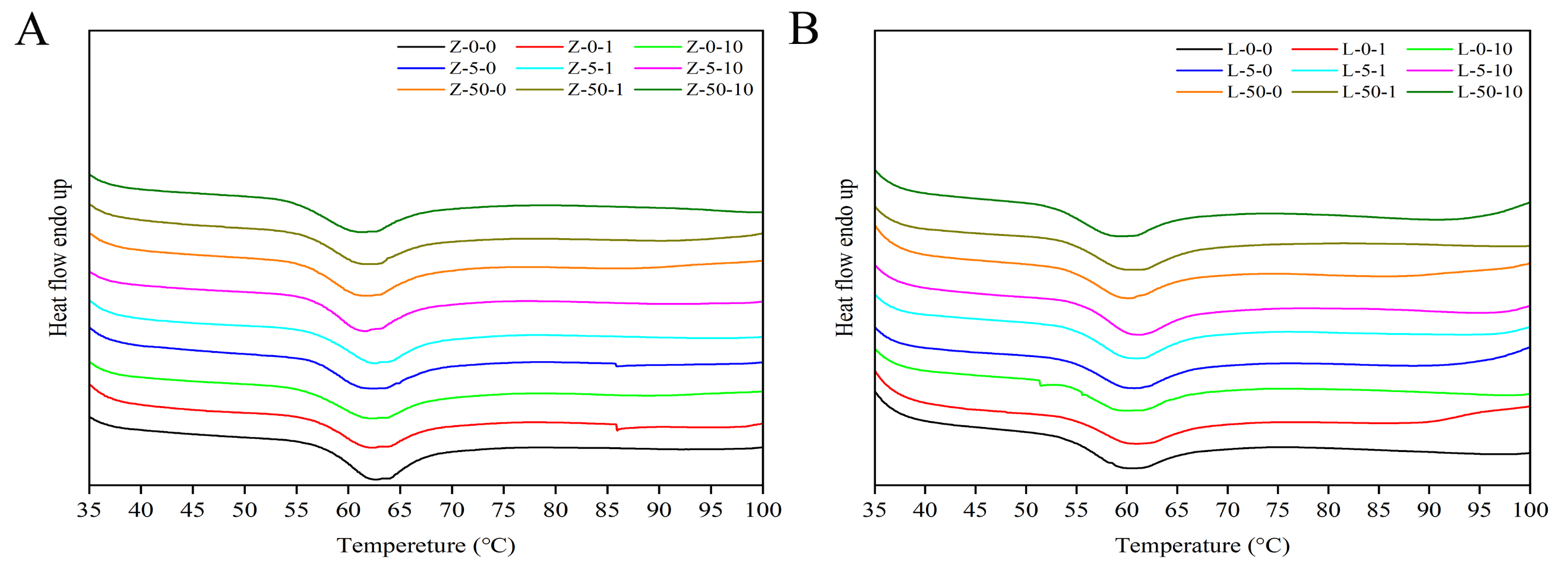


Supplementary Figure 3 DSC curves of starch in the grains of wheat cultivars under Cd stress and exogenous MeJA treatment. A and B represent the DSC curves of ZY and LM23, respectively.

Supplementary Table 1 The thousand-grain weight and grain starch content of wheat cultivars under Cd stress and exogenous MeJA treatment.

| Treatments | TGW (g) | TS (%) | AC (%) | Change (%)  TGW | Change (%)  TS | Change (%)  AC |
| --- | --- | --- | --- | --- | --- | --- |
| Z-0-0 | 32.82±0.86a | 75.70±0.24a | 18.45±0.14cd | — | — | — |
| Z-0-1 | 24.64±016d | 74.43±0.30b | 19.23±0.13b | -24.92 | -1.68 | 4.23 |
| Z-0-10 | 24.33±0.35d | 72.83±0.14cd | 18.35±0.07c | -25.87 | -3.79 | -0.54 |
| Z-5-0 | 21.81±0.49e | 68.69±0.11g | 20.87±0.04a | -33.55 | -9.26 | 13.12 |
| Z-5-1 | 25.62±0.13cd | 71.79±0.14e | 18.98±0.04b | 17.47 | 4.51 | -9.06 |
| Z-5-10 | 25.72±0.18cd | 72.19±0.49de | 18.86±0.05bc | 17.93 | 5.1 | -9.63 |
| Z-50-0 | 27.52±1.45ab | 73.14±0.24c | 20.77±0.51a | -16.15 | -3.38 | 12.57 |
| Z-50-1 | 28.37±1.43b | 74.88±0.21b | 20.57±0.30a | 3.09 | 2.38 | -0.96 |
| Z-50-10 | 20.23±0.04e | 69.43±0.13f | 20.65±0.48a | -26.49 | -5.07 | -0.58 |
| L-0-0 | 34.97±0.47a | 75.27±0.20a | 18.05±0.08bc | — | — | — |
| L-0-1 | 30.61±0.18b | 75.03±0.09a | 18.56±0.05ab | -12.47 | -0.32 | 2.83 |
| L-0-10 | 23.90±0.29f | 72.86±0.10d | 17.09±0.47c | -31.66 | -3.2 | -5.32 |
| L-5-0 | 27.07±0.03de | 73.52±0.13c | 19.57±0.27a | -22.59 | -2.32 | 8.42 |
| L-5-1 | 28.12±0.74cd | 74.82±0.15ab | 19.08±0.46ab | 3.88 | 1.77 | -2.5 |
| L-5-10 | 26.77±0.01e | 73.33±0.23cd | 18.81±0.74ab | -1.11 | -0.26 | -3.88 |
| L-50-0 | 30.17±0.10b | 74.40±0.33b | 19.76±0.99a | -13.73 | -1.16 | 9.47 |
| L-50-1 | 31.26±0.86b | 74.92±0.06a | 18.73±0.10ab | 3.61 | 0.7 | -5.21 |
| L-50-10 | 28.52±0.44c | 71.94±0.43e | 19.03±0.47ab | -5.47 | -3.31 | -3.69 |

TGW, thousand grain weight; TS, total starch content; AC, amylose content. The percentage increase or decrease was calculated relative to the corresponding control: Z/L-0-1, Z/L-0-10, Z/L-5-0, and Z/L-50-0 were compared with Z/L-0-0; Z/L-5-1 and Z/L-5-10 were compared with Z/L-5-0; Z/L-50-1 and Z/L-50-10 were compared with Z/L-50-0. Different letters above the bars indicate significant differences (LSD; *p* < 0.05) between the treatments of the same cultivar. Positive and negative values indicate increases and decreases relative to the corresponding control, respectively.

Supplementary Table 2 Effects of Cd stress and exogenous MeJA treatment on the Relative intensity and FTIR of wheat grain starch.

| Treatments | Relative intensity (%) | 1022/995 cm^−1^ | 1045/1022 cm^−1^ | Change (%)  Relative intensity | Change (%)  1022/995 cm^−1^ | Change (%)  1045/1022 cm^−1^ |
| --- | --- | --- | --- | --- | --- | --- |
| Z-0-0 | 22.70±0.43a | 0.872±0.007a | 0.661±0.003cd | — | — | — |
| Z-0-1 | 21.91±0.77a | 0.849±0.025abc | 0.644±0.029d | -3.48 | -2.64 | -2.57 |
| Z-0-10 | 22.27±0.17a | 0.842±0.032abc | 0.665±0.014cd | -1.89 | -3.44 | 0.61 |
| Z-5-0 | 15.26±0.48d | 0.812±0.008bc | 0.668±0.007cd | -32.78 | -6.88 | 1.06 |
| Z-5-1 | 16.78±0.08c | 0.821±0.020abc | 0.671±0.009bcd | 9.96 | 1.11 | 0.45 |
| Z-5-10 | 17.95±0.41bc | 0.808±0.012bc | 0.679±0.005abc | 17.63 | -0.49 | 1.65 |
| Z-50-0 | 17.54±0.51bc | 0.797±0.021c | 0.712±0.002a | -22.73 | -8.6 | 7.72 |
| Z-50-1 | 17.67±0.13bc | 0.831±0.032abc | 0.702±0.001ab | 0.74 | 4.27 | -1.4 |
| Z-50-10 | 19.27±0.20b | 0.859±0.001ab | 0.711±0.002a | 9.86 | 7.78 | -0.14 |
| L-0-0 | 20.38±0.04bc | 0.814±0.005c | 0.711±0.003f | — | — | — |
| L-0-1 | 17.59±0.04e | 0.845±0.004ab | 0.733±0.012ef | -13.69 | 3.81 | 3.09 |
| L-0-10 | 16.85±0.46e | 0.842±0.005ab | 0.756±0.002de | -17.32 | 3.44 | 6.33 |
| L-5-0 | 19.70±0.34c | 0.836±0.005abc | 0.784±0.002cd | -3.34 | 2.7 | 10.27 |
| L-5-1 | 20.45±0.19bc | 0.848±0.010ab | 0.798±0.003bc | 3.81 | 1.44 | 1.79 |
| L-5-10 | 22.38±0.32a | 0.858±0.017a | 0.82±0.019b | 13.6 | 2.63 | 4.59 |
| L-50-0 | 18.70±0.13d | 0.862±0.016a | 0.858±0.016a | -8.24 | 5.9 | 20.68 |
| L-50-1 | 20.84±0.42b | 0.828±0.001bc | 0.760±0.010de | 11.44 | -3.94 | -11.42 |
| L-50-10 | 21.68±0.34a | 0.842±0.009ab | 0.883±0.004a | 15.94 | -2.32 | 2.91 |

The percentage increase or decrease was calculated relative to the corresponding control: Z/L-0-1, Z/L-0-10, Z/L-5-0, and Z/L-50-0 were compared with Z/L-0-0; Z/L-5-1 and Z/L-5-10 were compared with Z/L-5-0; Z/L-50-1 and Z/L-50-10 were compared with Z/L-50-0. Different letters above the bars indicate significant differences (LSD; *p* < 0.05) between the treatments of the same cultivar. Positive and negative values indicate increases and decreases relative to the corresponding control, respectively.

Supplementary Table 3 The effect of Cd stress and exogenous MeJA on wheat starch solubility, swelling power, syneresis, and light transmittance.

| Treatments | SOL (%) | SP (%) | FTS (%) | LT (%) | Change (%)  SOL | Change (%)  SP | Change (%)  FTS | Change (%)  LT |
| --- | --- | --- | --- | --- | --- | --- | --- | --- |
| Z-0-0 | 12.83±0.50ab | 18.33±0.55a | 47.18±0.79a | 63.75±0.75a | — | — | — | — |
| Z-0-1 | 10.83±0.83c | 17.47±0.40c | 45.68±0.37ab | 61.15±0.05b | -15.59 | -4.69 | -3.18 | -4.08 |
| Z-0-10 | 9.33±0.0.33d | 17.53±0.47c | 44.71±0.82b | 55.60±0.50c | -27.28 | -4.36 | -5.24 | -12.78 |
| Z-5-0 | 12.00±0.33bc | 17.76±0.41abc | 40.03±0.49c | 55.25±0.85c | -6.47 | -3.11 | -15.15 | -9.65 |
| Z-5-1 | 12.84±0.67ab | 18.25±0.32ab | 38.20±0.25d | 54.95±1.35c | 7 | 2.76 | -4.57 | -1.17 |
| Z-5-10 | 11.77±0.10bc | 17.42±0.46bc | 39.73±0.54cd | 52.90±0.90d | -1.92 | -1.91 | -0.75 | -4.25 |
| Z-50-0 | 13.89±0.51a | 17.57±0.05bc | 41.45±0.24c | 54.70±0.90c | 8.26 | -4.15 | -12.14 | -0.45 |
| Z-50-1 | 13.50±0.50a | 17.64±0.37abc | 34.47±0.87e | 55.60±0.50c | -2.81 | 0.4 | -16.84 | 5.1 |
| Z-50-10 | 11.17±0.17c | 17.63±0.22abc | 39.84±0.72cd | 55.80±1.00c | -19.58 | 0.34 | -3.88 | 2.01 |
| L-0-0 | 13.53±0.32ab | 14.80±0.33abc | 47.60±0.46a | 69.35±0.45a | — | — | — | — |
| L-0-1 | 11.26±0.89cde | 14.12±0.03c | 45.44±0.93bc | 54.30±0.10b | -16.78 | -4.59 | -4.54 | -2.69 |
| L-0-10 | 12.74±0.92abc | 14.59±0.21abc | 46.67±0.81ab | 54.40±0.90b | -5.84 | -1.42 | -1.95 | -21.56 |
| L-5-0 | 12.7±0.20abc | 14.71±0.25abc | 43.69±0.81cd | 53.55±0.55bc | -6.13 | -0.61 | -8.21 | -1.38 |
| L-5-1 | 10.9±0.27de | 14.78±0.33abc | 42.43±0.81de | 51.20±0.20d | -14.17 | 0.48 | -2.88 | -5.88 |
| L-5-10 | 14.09±0.24a | 15.05±0.31ab | 41.61±0.50e | 52.10±0.60d | 10.94 | 2.31 | -4.76 | -2.71 |
| L-50-0 | 12.36±0.10bcd | 14.26±0.22bc | 44.89±0.95bc | 52.05±0.25d | -8.65 | -3.65 | -5.69 | 1.66 |
| L-50-1 | 11.72±0.65cde | 14.67±0.53ab | 42.47±0.94de | 51.15±0.15d | -5.18 | 2.88 | -5.39 | -1.82 |
| L-50-10 | 10.79±0.47e | 15.20±0.31a | 43.47±0.41cde | 52.5±0.60cd | -12.7 | 6.59 | -3.16 | 0.86 |

SOL, solubility; SP, swelling power; FTS, freeze–thaw stability; LT, Light transmittance. The percentage increase or decrease was calculated relative to the corresponding control: Z/L-0-1, Z/L-0-10, Z/L-5-0, and Z/L-50-0 were compared with Z/L-0-0; Z/L-5-1 and Z/L-5-10 were compared with Z/L-5-0; Z/L-50-1 and Z/L-50-10 were compared with Z/L-50-0. Different letters above the bars indicate significant differences (LSD; *p* < 0.05) between the treatments of the same cultivar. Positive and negative values indicate increases and decreases relative to the corresponding control, respectively.
